# Individuals in space: personality-dependent space use, movement and microhabitat use facilitate individual spatial niche specialization

**DOI:** 10.1007/s00442-019-04365-5

**Published:** 2019-03-02

**Authors:** Annika Schirmer, Antje Herde, Jana A. Eccard, Melanie Dammhahn

**Affiliations:** 1 Animal Ecology, Institute for Biochemistry and Biology, University of Potsdam, Maulbeerallee 1, 14469 Potsdam, Germany; 2 Plant Ecology and Nature Conservation, Institute for Biochemistry and Biology, University of Potsdam, Potsdam, Germany; 3 Department of Animal Behaviour, University of Bielefeld, Bielefeld, Germany

**Keywords:** Ecological niche, Inter-individual differences, Intraspecific competition, Movement ecology, Small mammal

## Abstract

Personality-dependent space use and movement might be crucially influencing ecological interactions, giving way to individual niche specialization. This new approach challenges classical niche theory with potentially great ecological consequences, but so far has only scarce empirical support. Here, we investigated if and how consistent inter-individual differences in behavior predict space use and movement patterns in free-ranging bank voles (*Myodes glareolus*) and thereby contribute to individual niche specialization. Individuals were captured and marked from three different subpopulations in North-East Germany. Inter-individual differences in boldness and exploration were quantified via repeated standardized tests directly in the field after capture. Subsequently, space use and movement patterns of a representative sample of the behavioral variation (n = 21 individuals) were monitored via automated VHF telemetry for a period of four days, yielding on average 384 locations per individual. Bolder individuals occupied larger home ranges and core areas (estimated via kernel density analyses), moved longer distances, spatially overlapped with fewer conspecifics and preferred different microhabitats based on vegetation cover compared to shyer individuals. We found evidence for personality-dependent space use, movement, and occupation of individual spatial niches in bank voles. Thus, besides dietary niche specialization also spatial dimensions of ecological niches vary among individuals within populations, which may have important consequences for ecological interactions within- and between species.

**Electronic supplementary material:**

The online version of this article (10.1007/s00442-019-04365-5) contains supplementary material, which is available to authorized users.

## Introduction

Understanding patterns, determinants and consequences of inter-individual variation within populations is a central theme in current ecological research (Bolnick et al. 2011; Dall et al. 2012; Sih et al. 2012; Wolf and Weissing 2012). Individuals of natural populations often occupy only part of the species’ ecological niche (Bolnick et al. 2003; Stamps and Groothuis 2010; Hart et al. 2016) and that has given way to the idea of individual niche specialization (Bolnick et al. 2003; Araújo et al. 2011). Individual niche specialization challenges classical niche theory (e.g., summarized in Chase and Leibold 2003), which has traditionally treated all individuals of a species as uniform regarding their ecological requirements and behavior (e.g., Chesson 2000; Adler, et al. 2007; Levine and HilleRisLambers 2009; Letten et al. 2017). Particularly, consistent inter-individual differences, e.g., animal personality (Gosling 2001), were suggested to affect important ecological processes and to generate spatio-temporal variability that influences individuals’ interactions with biotic and abiotic factors (Webster et al. 2009; Bolnick et al. 2011; Brodersen et al. 2011; Wolf and Weissing 2012; Pearish et al. 2013; Best et al. 2015; Holtmann et al. 2017). Since interactions are essential in forming the ecological niche of an individual, the acknowledgement of consistent differences among individuals, and therefore the occupation of individual niches that together form the whole species niche should be imperative (Bolnick et al. 2003). Such segregation into individual niches should decrease intraspecific competition and support the maintenance of variation in natural populations (Bolnick et al. 2003, 2011; Wolf and Weissing 2012).

Many ecological interactions, both within- and between species, are mediated by spatio-temporal variation of habitat use (e.g., Chappell 1978; Werner et al. 1981; Boon et al. 2008; Fischer and Schröder 2014; Owen-Smith 2015). Consequently, individual differences in movement and space use are key components of an individual’s ecological niche. However, so far, ecological research has mainly focussed on individual specialization in diet (Bolnick et al. 2003). Little is still known on how differential movement and space use might facilitate individual niche segregation. Therefore, the main aim of this study was to test whether consistent inter-individual differences in behavior predict space use and movement patterns in natural habitats thereby contributing to individual niche specialization.

Many classical movement studies treat conspecific individuals as ecologically and behaviorally equivalent, even though evidence for individual differences is apparent in many of them (Liro and Szacki 1987; Austin et al. 2004; Nathan et al. 2008; Hawkes 2009; Beest et al. 2014). Theoretical concepts, on the other hand, have taken individual variability in states into account and highlighted its importance for explaining intraspecific variation in movement patterns as well as the discrepancy between observed and expected behavior based on optimality approaches (Nathan et al. 2008; Jeltsch et al. 2013; Nilsson et al. 2014; Spiegel et al. 2017). Empirical studies testing predictions of these theoretical frameworks are recently emerging, suggesting that personality-dependent movement and space use should be incorporated into movement ecology studies (Chapman et al. 2011; Harrison et al. 2015; Spiegel et al. 2015). As pointed out by Spiegel et al. (2015), the majority of those studies incur a problem of non-independency because they base their personality quantification directly on the movement data. Moreover, most studies only observed resulting space use patterns of different behavioral types, but the underlying movement pattern might be an equally important factor shaping the spatial dynamics of natural populations. Here, we, therefore, independently quantified consistent individual differences in behavior, using standardized experimental procedures, and movement and space use patterns, using automated radio-tracking under natural conditions.

Movement in general can be separated into three distinctive types, foraging, dispersal and migration, depending on their spatio-temporal scale (Nathan et al. 2008; Clobert 2012; Jeltsch et al. 2013). Up till now, most research regarding the influence of individual differences on movement has focused on dispersal, i.e., large-scale movement. In general, more aggressive, bolder or more explorative individuals tend to disperse and cover larger dispersal distances compared to less aggressive, shy or less explorative individuals (Cote et al. 2010). For example, in western bluebirds (*Sialia mexicana*), the aggression of individuals positively affected their probability to colonize new areas, which supported a rapid range expansion of that species at the edges of its distribution (Duckworth and Badyaev 2007; Duckworth and Price 2008). Similarly, dispersing delicate skinks (*Lampropholis delicate*) had consistently higher levels of aggression compared to their resident conspecifics (Michelangeli et al. 2017), fast-exploring individuals of wild great tits (*Parus major*) dispersed further than their slow-exploring conspecifics (Dingemanse et al. 2003), and bolder individuals of the Trinidad killifish (*Rivulus hartii*) had larger dispersal distances than shy conspecifics (Fraser et al. 2001).

Besides extensive research on personality-dependent dispersal, only few studies focused on local movement types, even though those small-scale movements are of key importance regarding ecological interactions and the forming of individual niches (Kobler et al. 2009; Pearish et al. 2013; Best et al. 2015; Farine et al. 2015; Spiegel et al. 2015). These studies indicate that movement within a habitat is also influenced by individual differences. In sleepy lizards (*Tiliqua rugosa*), for example, boldness predicted home range size and aggression influenced the intensity of use of habitat areas (Spiegel et al. 2015). Furthermore, individual differences in behavior affected spatial distribution of individuals across microhabitats leading to a non-random distribution of behavioral types. For example, in pikes (*Esox lucius*) individuals consistently preferred different densities of vegetation cover in a lake and thereby occupied different areas which enabled the identification of different behavioral types (Kobler et al. 2009). This behavioral type–environment correlation, where certain behavioral types are more frequently found in specific environments, could also be shown for a natural population of sticklebacks: individuals that emerged from a refuge faster were more likely to be in shoals with other sticklebacks (Pearish et al. 2013). Behavioral type–environmental correlations, therefore, can refer to the social environment as well (Best et al. 2015; Farine et al. 2015). Resulting from this behavioral type–environment correlation, both in terms of abiotic, biotic or social environment, the spatial distribution of individuals is non-random.

A non-random distribution of behavioral types within a population’s habitat in turn influences the spatial overlap of these types. Consequently, the probability of individuals of similar behavioral type interacting, directly or indirectly, is much higher due to their spatial proximity and the use of similar resources. Individual differences, therefore, also result in non-random interactions between individuals (Pruitt and Ferrari 2011; Wolf and Weissing 2012; Pruitt and Modlmeier 2015). Quantifying and considering individual differences of neighboring individuals in natural populations is, thus, crucial to understand how these individuals interact in space and time. Furthermore, both restricted interactions and the experience of different environmental conditions should be two key aspects of individual niche specialization and the shaping of ecological communities (Bolnick et al. 2003, 2011). Empirical research testing these potential relationships is scarce, mainly due to challenges of quantifying behavioral variation of a set of neighboring individuals, while simultaneously tracking their movements through space.

Small mammals play a key role in ecosystems, being foragers/consumers themselves, as well as being highly susceptible to ground and avian predation. Many small rodents, such as mice and voles, are highly dependent on the characteristics of their habitat to minimize predation, preferring heterogeneous habitats consisting of woody vegetation combined with grassy areas, like woodlands, shrubs, hedges and meadows (Hansson 1978; Adler 1985; Hansson 1999; Fischer and Schröder 2014) that offer cover from predation. They are sensitive to even small changes in habitat structure (e.g., cover) and this sensitivity can drive population dynamics and density fluctuations as well as foraging movements of individuals (Jędrzejewski et al. 1993; Cook et al. 2004; Alain et al. 2006; Lee and Rhim 2016). Small mammals, therefore, provide a suitable study system because individuals spatially interact, they are trackable in sufficient numbers, show quantifiable consistent differences in behavior, and inhabit vegetation structures which can be quantified as proxy of predation risk and habitat quality (Eccard et al. 2008). Here, we used bank voles (*Myodes glareolus*) since personality traits like risk-taking, activity and exploration can be easily quantified under laboratory conditions (Korpela et al. 2011; Šíchová et al. 2014). Further, bank voles are sensitive to temporal changes in risk and adjust their behavior accordingly (Liesenjohann et al. 2011; Hoffmann et al. 2018).

In detail, we investigated the following hypotheses: (1) free-ranging bank voles show measurable consistent inter-individual differences in movement-related behaviors. We predicted individuals to quantitatively differ in boldness and exploration, i.e., these behaviors to be repeatable. (2) Inter-individual differences in movement-related behaviors predict inter-individual differences in space use and movement. Since behavioral traits, such as boldness and exploration, have been shown to be related to home range size, movement within a habitat, and interspecific interactions (e.g., Pruitt and Ferrari 2011; Wolf and Weissing 2012; Spiegel et al. 2015), they should also be key traits influencing the spatial patterns of a highly predated species such as bank voles. We predicted bolder and more explorative individuals to have larger home ranges, to have larger core areas, to move longer distances, and to spatially overlap more with conspecifics than less bold and explorative individuals. (3) Inter-individual differences in behavior are associated with differences in microhabitat use. Since boldness predicts risk-taking during foraging (e.g., Dammhahn and Almeling 2012) and bank voles adjust their risk-taking to perceived predation risk under varying levels of cover (Eccard et al. 2008), we expected home ranges and core areas of shy individuals to be characterized by higher maximum vegetation height and ground cover than those of bold individuals.

## Methods

### Study animals

Bank voles (Cricetidae, *M. glareolus*) are widely distributed throughout Eurasia and occupy heterogeneous habitats consisting of woody vegetation combined with grassy areas, like woodlands, shrubs, hedges and meadows (Mazurkiewicz and Rajska-Jurgiel 1987; Hansson 1999). The diet includes leaves, roots, seeds, fruits, and grass complemented with insects and other animal-based food (Gębczyńska 1976; Ostfeld 1985). The activity rhythm of bank voles is polyphasic with obvious activity peaks during twilight hours but additional activity bouts throughout the whole day occur (Baumler 1975; Wójcik and Wolk 1985). Populations fluctuate, with a cycle length of 3–5 years, with high densities in peak years, of up to 150 individuals per ha, followed by population crashes in the subsequent year (Ylönen et al. 1988; Korpela et al. 2011). The social organization is characterized by female territoriality, which is especially pronounced during the breeding season (April–October) when females reduce their home ranges and increase their exclusivity (Koskela et al. 1997, 2000; Ylönen and Horne 2002). Males are not territorial and usually spatially overlap several female territories (Mazurkiewicz and Mazurkiewicz 1971; Andrzejewski and Mazurkiewicz 1976; Ostfeld 1985).

### Study sites

The study was conducted on five study sites in the *AgroScapeLabs*, a joint research platform of the Berlin-Brandenburg Institute for Biodiversity Research (https://www.bbib.org/home.html), in North-West Brandenburg, Germany (53°21′56.2″N, 13°48′17.3″E). This area is characterized by intensively used large agricultural fields which are pierced by small unused areas that serve as refuges for the local biodiversity, like hedges and fallow lands. We selected areas of fallow lands as study sites because they are expected to host a variety of rodent species and are confined areas due to their restriction by agricultural fields. The vegetation of these sites was always heterogeneous consisting of grassy areas, streaked with nettles and bushes and a few trees. The most common plants were nettles (*Urtica* spp.) and horehound (*Ballota* spp.).

### Capture–mark–recapture

We captured animals with Ugglan live traps (Grahnab Sweden, Special no. 2) between August and November 2016. At each study site, 55 traps were set up in a trapping grid with approximately 10-m distance between traps, (covering 0.31–0.49 ha). Trapping grids remained in place until the whole experimental run, including trapping, individual difference tests, VHF tracking and recapture of collared individuals (see below), on the respective site was over. Traps were baited with rolled oats and apples. Before trapping commenced for the first time on a study site, the traps were pre-baited for 24 h, after which the traps were activated and the initial trapping session started. Upon initial capture, each individual was marked with an individual fur cut, weighed, sexed, and determined to species. Trapping continued until > 95% of the captured animals were marked. At the five study sites, we captured between 75 and 103 rodents, representing a density of 151–260 rodents ha^−1^, of which between 20 and 75 were bank voles (40–162 ha^−1^). Other species included striped field mouse (*Apodemus agrarius*), common vole (*Microtus arvalis*), field vole (*M*. *agrestis*), yellow-necked mouse (*Apodemus flavicollis*) and wood mouse (*A*. *sylvaticus,* species are presented with declining abundances). Individuals that entered the individual difference test (see below) were marked permanently with a passive integrated transponder (PIT, Euro I.D., trovan^®^ ID100) after their first individual difference test for identification in the second test when fur marks had partly grown back.

### Individual difference test

We tested for consistent between-individual differences on all five trapping grids using a standardized behavioral test in the field. Upon first recapture, each individual entered this test, excluding juveniles (< 17 g body mass). The test setup consisted of an opaque plastic pipe (10.5 × 32 cm) with a swing door at each end, attached to a round arena (diameter 1.30 m, 30 cm height; Appendix Fig. A1). Captured individuals were placed in their respective trap in front of the test setup and left to enter it on their own accord. The setup excludes the need to handle individuals before testing, precluding possible influences of handling stress on behavioral expression during the test. The latency to leave the trap was noted, as a possible emergence test, but later discarded due to lack of repeatability (see below). Tests were conducted directly upon capture of individuals within their natural habitat at one location on each study site, without translocating them. During the test individuals were exposed to natural environmental conditions. We chose comparable test locations at each study site and restricted testing to days with favorable weather conditions (low wind speed, no rain).

The setup is a combination of two established laboratory tests for individual differences in behavior of rodents (Archer 1973; Herde and Eccard 2013), the dark–light test and the open-field test (Appendix Fig. A1). The dark–light test measures willingness of individuals to enter an unknown and potentially risky area. The dark and ceiled compartment (pipe) is assumed to represent cover and safety, while the open and light arena represents an unknown, potentially risky area. By direct observation, we quantified the latencies to enter the arena with the head and with the full body (excluding tail) in seconds. If an individual did not enter the arena within 300 s, the latency was set to 300 s and the individual was gently forced out of the pipe into the arena by hand. Besides being the light compartment in the first test part of the test, the open arena also represents the classic open field setup where the individual behavior in a novel environment is quantified by assuming different levels of perceived risk in different arena parts. The border area, where the wall resembles cover, is assumed to be safe while the exposed middle part of the arena represents a high-risk area. Middle and border parts were divided in a way that they covered the same surface area. Furthermore, the arena was virtually divided into 16 sections to enable quantification of the exploration effort of each individual. We quantified the following variables via direct observation over the test period of 300 s: (1) the latency to enter the middle area for the first time (full body excluding tail), (2) the number of different sections entered, (3) the number of crossings into the middle area, (4) the number of jumps, and (5) the proportion of time spent active, which was assessed instantaneously every 10 s. Individuals were defined as active when they were either running, jumping, grooming or sitting and moving their head scanning the surroundings. This test part started immediately after individuals exited the dark compartment of the previous test part and a re-entering was made impossible by closing the swing door. Tests were repeated upon recapture of the individual (1–7 days later) at least twice for 62 individuals.

### Automated radio telemetry

On three of the five trapping grids with the highest densities (151, 198 and 222 rodents ha^−1^), we assessed space use and movement distances. At these sites, we equipped a total of 21 individuals (6–8 per site) with VHF radio transmitters (1.1 g, BD-2C, Holohil Systems Ltd., Canada) applied on a collar and tracked them via automated radio telemetry for a total of 4 days. We selected only individuals that were residential (i.e., recaptured > 2 times) and had a body mass that allowed the carrying of a transmitter without exceeding a ratio of transmitter to body mass of 0.05. Females that were in the last stages of gestation, based on visual inspection, and expected to give birth within the tracking period were excluded, which resulted in 9 females being tracked and 12 males. Substantial effort was made to recapture animals to remove collars after data collection, including increasing the number of traps, using different types of live traps and bait and intensive trapping over several weeks.

The tracking system contained a grid of eight omnidirectional antennas (GP 150 Winkler-Spezialantennen, Annaberg, Germany), surrounding the trapping grid at ground level, and two automated multi-channel receiving units (ARU, JDMC Corp, Illinois, US), each one connected to four of the antennas (Appendix Fig. A2). The ARUs recorded noise and signal strengths of the respective transmitter frequency at each connected antenna seven times in a row for 24 s every 20 min, i.e., ca. 96 times per day. We used the median signal strength of seven repeats per antenna for further calculations (see below) to reduce errors from atmospheric disturbances or sudden movements of the animal which can strengthen or weaken single signal pulses. Signal strength was summed up over each side of the arbitrary telemetry grid (3 antennas per side). The proportion of signal strength among the sides was used to calculate an isoline through the grid in both *x* and *y* direction, yielding *x–y* coordinates for each telemetry fix. Immediately before the tracking of animals commenced, we calibrated isolines based on known location points using rotating test transmitters. Accuracy of location calculation was ca. 10 m for each location point. Obvious outliers (distances of > 50 m to other location points due to strong wind events) in location data were removed from the data set.

### Spatial analyses

Home range size and overlap were based on kernel density analyses of 95% (defined as whole home range) and 50% (defined as core area) of location points. Home ranges, overlaps and movement distances were based on individual locations points of all 4 days of tracking. Spatial analyses were done with the R packages adehabitat (version 1.8.18), *adehabitatHR* (version 0.4.14) and *adehabitatLT* (version 0.3.21; Calenge 2006). We were only able to track the movement and space use of a subset of individuals present at a site, which might impair the analysis of spatial overlap. Therefore, we additionally calculated the mean trapping point of each individual as a proxy of its home range center from capture–mark–recapture data for all trapped bank voles. Subsequently, we quantified the number of mean trapping points located within the home ranges and core areas of tracked individuals. This analysis allowed us to assess spatial interactions between tracked individuals and all residential individuals present at each site. Mean trapping points of individuals were based on 4.33 ± 3.56 (mean ± SD) captures/individual. The number of mean trapping points per tracked home range and core area was extracted with the program QGIS (version 2.18.14).

### Microhabitat structure

Habitat characteristics were determined by measuring the maximum vegetation height (in cm) in a square meter around each trap location of each trapping grid. This maximum vegetation height correlates well with average vegetation height (M. Dammhahn, unpublished data). We choose maximum vegetation height as a habitat characteristic because at our study sites, microhabitats mainly varied between areas of grass and herbal vegetation of ca. 94.6 ± 76.4 cm height and small trees and bushes of ca. 151.9 ± 105.6 cm height (Fig. A3). We assumed that bank voles moving in microhabitats dominated by grass (low maximal vegetation height, high cover) are less susceptible to ground predation (impaired movement of larger predators through dense vegetation) but more susceptible to avian predation (better visibility for aerial predators). Bank voles moving under trees and bushes are assumed to be better protected from aerial predation but more susceptible to ground predators. Percentage of ground cover (10 cm from the ground) was calculated afterwards on the basis of photographs taken of the square meter around the trap locations. Based on the trapping grid, we interpolated local maximum vegetation height and local percentage of ground cover with an inverse weighing of the distance between points using QGIS (version 2.18.14). We visually verified these interpolations by the use of satellite images. We then extracted the interpolated value for the respective habitat characteristic for each telemetry fix of individuals. These values were used to calculate means of maximum vegetation height and average ground cover for the home range and core area of individuals. Although distribution and availability of food resources also affect movement and space use of small mammals (e.g., Morris 1997; Liesenjohann et al. 2011), we focussed here only on vegetation cover (i.e., a proxy of predation risk) due to logistic challenges of quantifying distribution and availability of all major components of the omnivorous diet of bank voles at the home range scale.

### Statistical analyses

#### Individual differences

Recorded variables were checked for consistency across test rounds by calculating repeatability according to Nakagawa and Schielzeth (2010) with the R package *rptR* (Version 0.6.405; Appendix Table A2). Latencies to emerge and investigate the arena were inverted for easier interpretation afterwards. Repeatable variables were then entered into a principal component analysis (PCA) with *oblimin* rotation to reduce the number of variables into meaningful components. All variables from the individual difference test were checked for suitability for a PCA by examination of the determinant of the correlation matrix, the Bartlett test, and the Kaiser–Mayer–Olkin (KMO) criterion (Field et al. 2012). We retained components with an Eigenvalue > 1 (Wold et al. 1987). We tested repeatability of PC components as described above.

Individual scores from the PCA were then entered into a Bayesian mixed-effects model with the experimental day (centered for the individual) as a fixed effect and individual as a random effect. This approach enabled us to account for differences in the time periods between consecutive tests (varying from 1 to 7 days, due to the unpredictability of recapturing individuals in a free-ranging population) and to control for the potential resulting variation of behavioral responses in the test setup (Hadfield et al. 2010; Cowles 2013; Marin and Robert 2014). We extracted linear unbiased predictions based on the Bayesian mixed-effects model because this approach results in less biased estimates (Hadfield et al. 2010) and used these estimates as quantitative measures for individual differences in further analyses. Furthermore, we calculated a Spearman rank correlation to test for an association between the two quantitative behavioral measures at the phenotypic level.

#### Individual differences and space use

To test our predictions, we ran linear mixed-effects models (LMMs) and generalized linear mixed-effects models (GLMMs) according to the underlying structure of the data. If feasible, data were transformed before the statistical modeling to achieve normality and a LMM was calculated. If a normal distribution could not be achieved for the respective response variable, GLMMs were conducted modeling the appropriate error structure of the data via the underlying distribution family and corresponding link function. All models were run with either the function *lmer* or *glmer* from the R package *lme4* (Version 1.1-12; Bates 2010).

Since individuals originated from different study sites (with varying population densities, vegetation differences, and differences in surrounding matrices), we included study site as a random factor, specified as random intercept, in each model. In general, home range sizes can vary with population density (e.g., Erlinge et al. 1990; Bond and Wolff 1999), but population density did not explain variation in home range size in our data set (Table A5). Therefore, we decided to control for slight differences in population density among sites within the random structure of our models.

Individual difference scores and sex of individuals were included as fixed effects in each model. Due to the small sample size, we could not include both behavioral scores in one model but run separate models with only one behavioral score instead. The proportion of explained variance by the fixed factors alone (marginal *R*^2^) and the fixed and random factors together (conditional *R*^2^) was estimated for each model according to Nakagawa and Schielzeth (2013). These values represent goodness-of-fit measures of GLMMs similar to the *R*^2^ value of generalized linear models (Johnson 2014; Nakagawa and Schielzeth 2013). The level of significance was set to *α* < 0.05. All calculations were done with the program R (Version 3.3.0, R Core Team 2016).

## Results

### Individual differences

All but two variables quantified in the individual difference test were repeatable over time (Appendix Table A2). Data reduction of repeatable variables via PCA rendered two meaningful components (Appendix Fig. A4a, Table A3) that cumulatively explained 78% of the variance in the data. On the first component, the latency to enter the middle area for the first time, the number of sections entered, the number of crossings into the middle area, and the proportion of time spent active had the highest loadings. The latency to investigate an unknown, open area and the latency to emerge with the full body into an unknown, open area had the highest loadings on the second component. Based on these loadings, the first component (explained variance: 52%) was interpreted as a measure of exploration and the second component (explained variance: 26%) as a measure of boldness (Appendix Fig. A4b). Higher values on the first component represent individuals with a higher number of crossings, sections explored, activity counts and shorter latencies to cross the central part of the arena, i.e., more explorative individuals. Higher values of PC2 correspond to shorter latencies to investigate and emerge into an unknown area, i.e., bolder individuals. Individual differences in both components were repeatable over time (PC1: *R* = 0.217, SE 0.122, 95% CI [0.003, 0.450], *p * = 0.023; PC2: *R* = 0.453, SE 0.125, 95% CI [0.191, 0.672], *p * = 0.001). The behavioral types of individuals selected for radio-tracking represent much of the populations’ variation in exploration and intermediate boldness types (Appendix Fig. A5). Exploration and boldness were not correlated at the phenotypic level (*S* = 4823, rho = − 0.16, *p* value = 0.22).

### Individual differences and space use

#### Home range size and distance moved

**Fig. 1:**
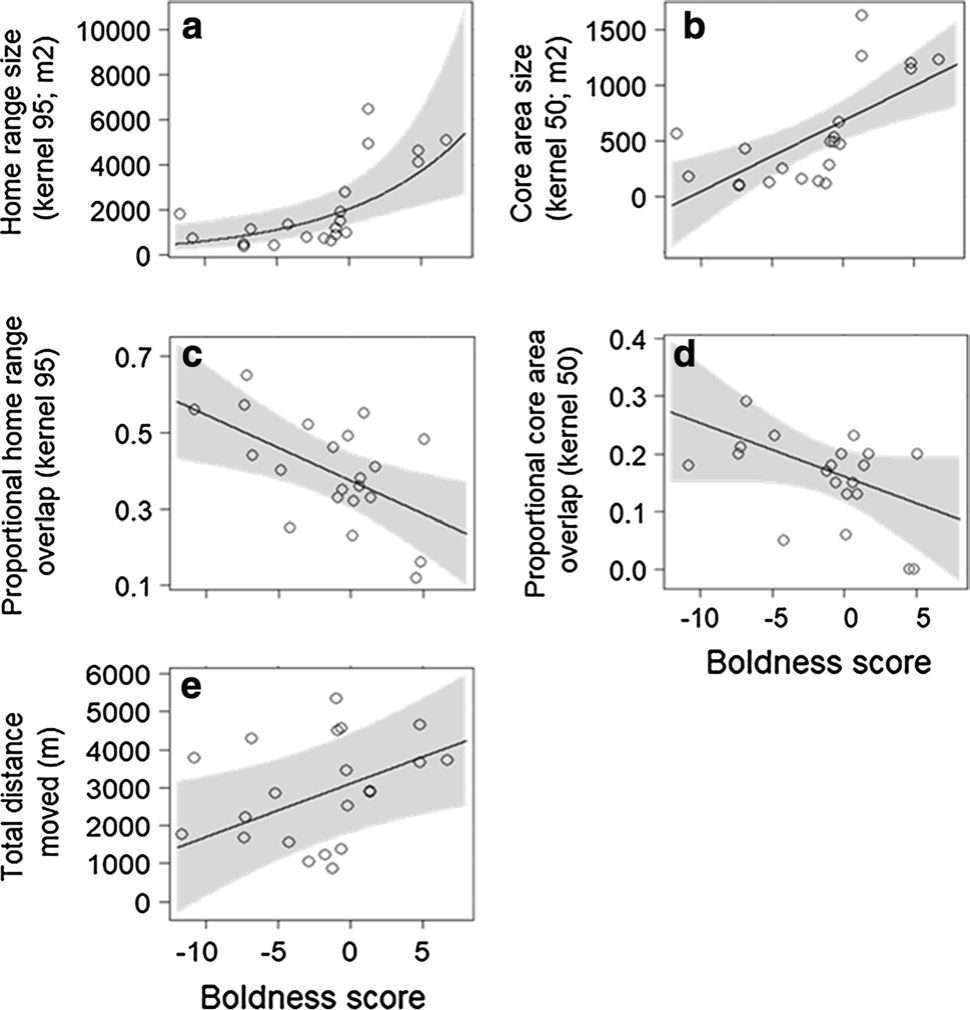
Effects of boldness on home range and core area size (**a**, **b**), spatial interactions (**c**, **d**), and movement distance (**e**) of 21 radio-tracked bank voles, *Myodes glareolus*. Represented are effects obtained from LMMs or GLMMs (line and shaded 95%-confidence intervals) and raw data of individuals (dots). Model effects were back transformed to the original data scale for visual representation if needed

Bolder bank vole individuals had larger home ranges, larger core areas and moved longer distances than shy individuals (Fig. 1a, b, e; Table 1). Males and females did not differ in either home range or core area size or total distance moved. Exploration scores did not explain variation in home range and core area size but more explorative animals moved shorter distances compared to their less explorative conspecifics (Fig. A6; Table 1). The random factor controlling for differences between study sites explained 9% of the variance in home range size and 0% in core area size (Table 1). In total, the mixed models explained 47% (home range size), 44% (core area size) and 67% (total distance moved) of the variance in the data (Table 1).

**Table 1:** Details and results of (G)LMMs on the effects of exploration and boldness on space use and movement patterns in bank voles, *Myodes glareolus* (*n* = 21)

Response	Personality score	Estimate	SE	*z/t-*value	*χ* ^2^	*df*	*p* value	*R*_m_	*R*_c_
Home range	Boldness	155.67	39.90	3.90	15.05	**1**	**< 0.001**	0.38	0.47
	Exploration	− 16.08	12.66	1. 27	1.55	**1**	0.214	0.32	0.30
Core area	Boldness	1.38	0.35	3.92	15.35	**1**	**< 0.001**	0.44	0.44
	Exploration	− 88.47	134.41	0.66	0.43	**1**	0.510	0.03	0.15
Total distance moved	Boldness	161.37	48.85	3.30	10.91	**1**	**0.001**	0.27	0.69
	Exploration	− 38.81	16.35	2.37	5.64	**1**	**0.018**	0.07	0.33
Intraspecific home range overlap	Boldness	− 0.02	0.01	3.12	9.71	**1**	**0.002**	0.37	0.49
	Exploration	0.00	0.00	1.91	3.65	**1**	0.056	0.10	0.38
Intraspecific core area overlap	Boldeness	− 0.04	0.02	2.13	4.55	**1**	**0.033**	0.13	0.13
	Exploration	0.01	0.01	1.15	1.32	**1**	0.281	0.06	0.06
No. of mean trapping points in home range	Boldeness	− 0.05	0.02	2.39	5.72	**1**	**0.017**	0.13	0.53
	Exploration	0.00	0.02	0.03	0.00	**1**	0.979	0.02	0.54
No. of mean trapping points in core area	Boldeness	− 0.08	0.03	2.46	6.07	**1**	**0.012**	0.13	0.64
	Exploration	0.03	0.03	0.79	0.62	**1**	0.432	0.04	0.68
Max. vegetation height in home range	Boldness	− 0.74	0.34	2.16	4.66	**1**	**0.031**	0.14	0.55
	Exploration	0.01	0.01	1.08	1.16	**1**	0.315	0.02	0.46
Max. vegetation height in core area	Boldness	− 0.51	7.65	0.50	0.25	**1**	0.615	0.21	0.70
	Exploration	0.03	0.35	0.31	0.01	**1**	0.927	0.06	0.09
Ground cover in home range	Boldness	0.61	0.30	2.01	4.05	**1**	**0.044**	0.19	0.23
	Exploration	0.05	0.11	0.47	0.22	**1**	0.638	0.03	0.26
Ground cover in core area	Boldness	0.06	0.61	0.09	0.01	**1**	0.928	0.01	0.01
	Exploration	0.08	0.21	0.40	0.16	**1**	0.692	0.09	0.09

The fixed factor sex never showed a significant influence and was therefore excluded from the representation. Home ranges refer to Kernel 95% and core areas to Kernel 50%

Statistically significant results are highlighted with bold font for *p* values

*SE* the standard error of the estimates, *z/t-value* the *z* and the *t* statistic, *df* degrees of freedom, family describes the distribution of the error structure of the model, link the associated link function, *R*_*m*_ marginal *R*^²^ value based on fixed factors, *R*_*c*_ conditional *R*^²^ value including the study site as a random factor

#### Intraspecific spatial overlap

Based on radio-tracking data, home ranges and core areas of bolder individuals overlapped less with conspecifics than home ranges and core areas of shyer individuals (Fig. 1c, d; Table 1). The exploration score had no effect on the spatial overlap patterns of home ranges or core areas in bank voles (Fig. A6; Table 1). Males and females did not differ in their overlap patterns of either home ranges or core areas. For the overlap of core areas, the mixed model explained 13% of the variance in the data and 49% for the overlap of home ranges; the random structure accounted for 0% in the core area models and 13% of the variance in the home range models (Table 1). Including spatial information on all residential individuals, these patterns were confirmed. Bolder individuals had less mean trapping points of conspecifics within their home ranges and core areas (Fig. A7a, b; Table 1). Sex or exploration did not affect the number of mean trapping points of conspecifics in home ranges and core areas. For core areas, the mixed model explained 64% of the variance in the data, 51% of that were added by the random effect; and for home ranges, 53% of the variance was explained with the random factor accounting for 40% (Table 1).

#### Vegetation height and ground cover

**Fig. 2:**
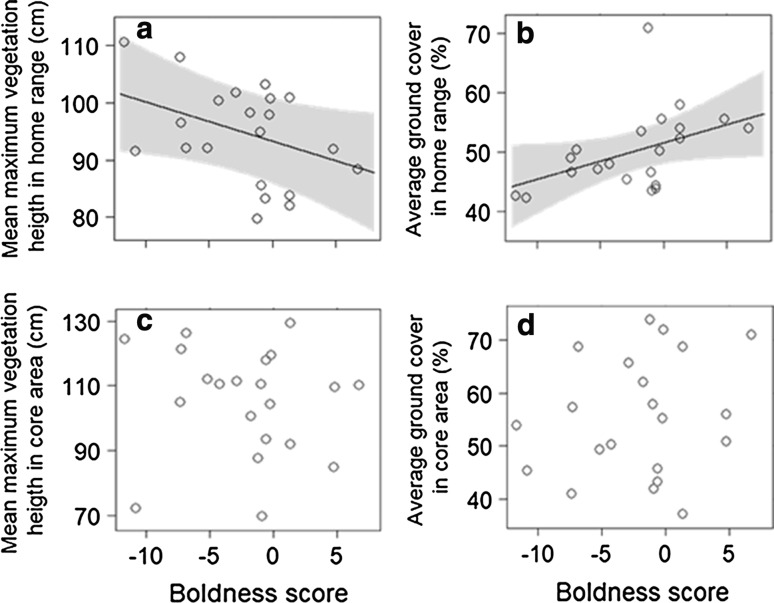
Effects of boldness on mean maximum vegetation and average ground cover of home ranges (**a**, **b**) and core areas (**c**, **d**) of 21 radio-tracked bank voles, *Myodes glareolus*. Represented are effects obtained from GLMMs (line and shaded area 95%-confidence interval) and raw data of individuals (dots). Model effects were back transformed to the original data scale for visual representation if needed

The maximum vegetation height of home ranges was on average between 80 cm and 111 cm and average ground cover was of 50%. The home ranges of bolder bank voles had a lower maximum vegetation height than those of their shyer conspecifics (Fig. 2 a; Table 1). In contrast, average percentage of ground cover was higher in home ranges of bolder as compared to shyer individuals (Fig. 2b; Table 1). For core areas, no difference in maximum vegetation height and percentage of ground cover could be detected between bold and shy bank voles (Fig. 2c, d; Table 1). Similarly, we did not detect a relationship between exploration score or sex and microhabitat characteristics of either home ranges or core areas. For the core areas the mixed model of the maximum vegetation height explained 70% of the variance in the data and 1% for amount of ground cover, random factors explained 49% and 0%, respectively. For the home ranges, the model regarding the maximum vegetation height explained 55% of the variance in the data and for the amount of ground cover 23%, random effects accounted for 41% and 4% of the explained variance (Table 1).

## Discussion

Combining intensive capture–mark–recapture and simultaneous automated radio-tracking of individuals with known behavioral phenotypes with small-scale assessment of microhabitat quality, we showed that consistent individual differences in boldness predicted intraspecific variation in home range and core area sizes, movement patterns and microhabitat use of free-ranging bank voles. Bolder animals occupied larger home ranges, spatially overlapped less with conspecifics, and used areas with higher ground cover and lower maximum vegetation height compared to shyer conspecifics. These results indicate a covariance of spatial niche components and consistent individual differences in behavior suggesting segregation of behavioral types into individual ecological niches.

### Consistent inter-individual differences in movement-related behaviors

Repeated behavioral tests of free-ranging bank voles revealed consistent inter-individual differences in boldness and exploration. Differences in both traits have been found to affect movement parameters in birds (Dingemanse et al. 2003), lizards (Spiegel et al. 2015) and fish (Fraser et al. 2001). In bank voles, however, boldness was a better predictor of space use than exploration. Contrary to findings of some other studies (e.g., Herde and Eccard 2013 in common voles; Bajer et al. 2015 in European green lizards) exploration and boldness did not correlate at the phenotypic level in bank voles. We tested individuals directly in the field after capture, which is a rare approach (see also Martin and Réale 2008; Dammhahn 2012; Best et al. 2015; Mella et al. 2016), and allowed the individual to enter the test apparatus voluntarily, which might facilitate disentangling of boldness-related and exploration-related behaviors (Carter et al. 2013; Perals et al. 2017).

### Personality-dependent space use and movement

We found a strong influence of boldness on home range size, core area size, and distances moved in bank voles. As predicted, bolder individuals occupied larger areas and moved longer distances compared to shy individuals, which is in accordance with previous studies on other species (Boon et al. 2008; Minderman et al. 2010; Spiegel et al. 2015). In American red squirrels (*Tamiasciurus hudsonicus*) inter-individual differences in activity and aggression affected the maximum trapping distance and the number of trap locations (Boon et al. 2008). In starlings (*Sturnus vulgaris*), areas explored in a novel environment test scaled positively with the size of individual home ranges obtained by radio-tracking (Minderman et al. 2010). In sleepy lizards (*T. rugosa*), both boldness and aggression influenced space use behavior (Spiegel et al. 2015) with boldness positively affecting the home range size, while aggression negatively impacted the intensity of use of areas close to the home range center.

A strong relationship between boldness, a predictor of risk-taking (e.g., Dammhahn and Almeling 2012), and spatial patterns in bank voles could be related to the general ecology of the species. In the following, we discuss several non-mutually exclusive explanations for this relationship.

First, the main source of mortality for voles, and most small mammals, is predation by ground and avian predators, rendering them a key species in natural food chains (Halle 1988; Jędrzejewski et al. 1993; Korpimäki 1993; Korpimaki et al. 1994; Gliwicz and Dabrowski 2008). Since boldness is a trait that is directly linked to mortality risk (Smith and Blumstein 2008), it should have a strong impact on space use patterns which influence the exposure to predators (see also discussion on microhabitat use below). As expected, shy individuals range over shorter distances and over smaller, potentially more familiar areas, which should reduce their predation risk.

Second, ranging further might enhance the chances of exploiting spatially dispersed and/or better-quality resources which might trade-off elevated mortality risk associated with this behavior, ultimately enabling the coexistence of individuals with different behavioral phenotypes in a population (Wolf and Weissing 2012). Larger home ranges and longer movement paths might, thus, increase access to spatially dispersed resources for bolder individuals.

Third, space use and movement of bold and shy individuals might reflect differential exploration styles between these behavioral types. Mettke-Hofmann et al. (2005) could show in parrots that differential exploration styles of resident (thorough explorers) and nomadic (superficial explorers) species influenced their choice of food; large proportion of the diet of thoroughly exploring parrots contained fruits and leaves; therefore, long-term available resources, while nomadic parrots preferred short-term available resources. Additionally, superficially exploring individuals are assumed to not deplete resources completely but rather shorten their average stay in, for example, a food patch and move on quickly to a new spot (Wolf et al. 2007). Therefore, these individuals might need to cover larger distances and consequently range over larger areas to acquire sufficient resources (Mazza et al. 2018). In contrast, shyer individuals cover less distance in the same amount of time, suggesting a more thorough exploration style. In that case, smaller distances and resulting smaller ranging areas might be sufficient in providing the necessary resources, because individuals know the area in which they move in great detail, enabling them to exploit all existing resources more optimally (Arvidsson and Matthysen 2016). Thorough exploration of an open-field arena was indeed negatively related to moved distances in the field in our data set. Whether this link between behavioral variation and space use does indeed reflect different exploration styles requires further testing.

Fourth, bold individuals might range further to fuel their elevated metabolism. In many species, boldness scales positively with metabolic rate and it has been shown that individuals with faster metabolic rates need more resources to satisfy their energetic requirements compared to shy individuals (Biro and Stamps 2008; Careau et al. 2008, 2009; Réale et al. 2010; Mathot and Dingemanse 2015). Since basal metabolic rate in bank voles is repeatable (Labocha et al. 2004), has a heritable component and is related to other performance traits (Sadowska et al. 2015), this hypothesis warrants further testing.

Bolder individuals also occupied larger and more exclusive core areas as evident from the spatial overlap patterns and the number of mean trapping points of residential individuals within core areas and home ranges, which might indicate stronger territoriality. The social system of the bank vole is solitary and characterized by strong female territoriality, while males have larger, overlapping ranges (Bujalska 1985; Gipps 1985). Females generally keep core areas exclusive and meet intruders with high levels of aggression (Koskela et al. 1997, 2000; Ylönen and Horne 2002). However, under high population density—such as at our study sites with 99 ± 61 individuals ha^−1^—female territoriality could break down (Ylönen et al. 1988). Interestingly, the relationship between boldness, core area size and exclusivity was not sex specific in our data. This pattern could mean monopolization of resources and decreased intraspecific competition might result from an increased competitive ability of bolder individuals in both sexes. With the data at hand, we cannot test whether bold individuals displace shy conspecifics or whether shy individuals avoid areas of bolder ones, but findings from other species often report behavioral syndromes between boldness and aggressiveness (Huntingford 1976; Sih et al. 2004) and bolder bank vole males are also dominant over shy individuals in encounters (Eccard, unpublished data).

Shyer individuals overlapped spatially with more conspecifics, both for the tracked home ranges as well as with mean trapping points of residential individuals, which might indicate that they are not able to keep their roaming areas exclusive and might be confronted with higher levels of intraspecific resource competition but on the other hand might have more and easier access to mating partners which could positively affect their reproductive success. Furthermore, under natural conditions, bank voles are in competition not only with themselves but also with other rodent species; therefore, even if shy individuals are confronted with higher levels of intraspecific competition, they might have an advantage over bold individuals in interspecific interactions, leading to the maintenance of both personality types in the population.

We could show that personality-dependent spatial overlap patterns lead to non-random intraspecific spatial interactions in bank voles. Whether this is a result of indirect exploitation competition, or of direct, aggressive intraspecific interactions, or a combination of both, cannot be answered with this study but might be an important avenue for future research.

### Personality-dependent occupation of microhabitats

At the core-area scale, there was no relationship between personality traits and microhabitat characteristics. Core areas represent the most frequently visited and used area of an individual, indicating home site, refuges or important food sources (Samuel et al. 1985; Seaman and Powell 1990). In the case of the bank vole, a species that occupies underground cavities where it builds its nest, stores food, and females raise their young (Braun and Dieterlen 2005), it is most likely that the core area represents the site where such cavities can generally be found. Bank voles are active during day and night with an activity bout every three–four hours (Ylönen et al. 1988; Braun and Dieterlen 2005) in between which they retreat into the nest. These aspects make the core areas highly valuable (i.e., fitness relevant) for bank voles and it might be equally important for both bold and shy individuals that the nest is located in the safest area of each individual’s home range. Indeed, core areas had both higher vegetation height and higher percentage of ground cover (Appendix Fig. A8) as compared to home ranges and, thus, represent areas that are greatly sheltered from both ground and avian predation.

The highest level of risk for a prey species, such as the bank vole, is encountered during the active phases when they are roaming above ground in search for food and mating partners (Braun and Dieterlen 2005). Therefore, it is not surprising that differences in the preferred vegetation cover can be found between bold and shy individuals at the home range level. Against our prediction and in contrast to other studies (Carrete and Tella 2010; Holtmann et al. 2017), bolder individuals more frequently used areas with high levels of ground cover and shy individuals were more frequently found in areas that potentially pose a higher predation risk. In our mixed habitat of grassland and shrub/tree islands, this means that bolder animals were using more grassy areas than shy animals (Fig. A3). This might further support a difference in competitive ability between the behavioral types leading to either the active displacement of shy individuals or competition avoidance, forcing shy individuals to settle in high-risk areas. In turn, shyer individuals might occupy smaller home ranges and move less to minimize their exposure in the risky roaming area leading to the observed space use patterns. Alternatively, it is also possible that individuals born in the areas of different levels of vegetation cover adjust their personality accordingly and that the environment shapes the personality rather than personality predicting the environment (Holtmann et al. 2017). Based on our data, we can not disentangle which of these two mechanisms drives the observed patterns. However, given similar preferences of behavioral types for core area microhabitats, juveniles of either bold or shy females appear to initially experience similar microhabitat conditions.

Personality-dependent preferred vegetation cover could also indicate susceptibility of behavioral types to different predators. Areas with low ground cover are usually paired with high maximum vegetation cover like trees and dense bushes on our study sites. Those vegetation characteristics limit the access of avian predators like, for example, common buzzards (*Buteo buteo*), which prefer hunting above open areas and are one of the most common predators of voles in Europe (Jędrzejewski and Jędrzejewska 1993; Norrdahl and Korpimäki 1995; Selas et al. 2007). For such predators, areas of low maximum vegetation height and more ground cover might be more accessible putting bolder individuals at higher risk compared to shyer individuals. Ground predators like foxes (*Vulpes vulpes*) and least weasel (*Mustela nivalis*), which are also common predators of bank voles (Jędrzejewski and Jędrzejewska 1993; Jędrzejewski et al. 1993; Kjellander and Nordström 2003), might, on the other hand, have an advantage in areas of low ground cover due to the higher exposure of prey individuals. Hence, shy individuals are likely more susceptible to ground predation based on their home range characteristics than bold individuals. Ultimately, the difference in home range vegetation cover might, therefore, indicate non-random predator–prey interactions for the behavioral types which could result in additional behavioral differences regarding predator avoidance and specialization of behavioral types to their microhabitat.

## Conclusion: personality and individual niche differentiation

Bank voles differ in their space use, movement and habitat choice according to their personality, resulting in non-random distributions of behavioral types within the habitat as well as non-random intraspecific spatial interactions. Depending on their behavioral type, individuals experience different levels of intraspecific competition over resources and occupy microhabitats of varying predation risk; hence, they occupy individual ecological niches.

Within-species variation in ecological niches could reduce intraspecific competition because individual behavioral types are less similar in their resources use and experience different main predators and, thus, face reduced exploitation competition for food- and predator-free area. Our results indicate that, in addition to individual dietary niche separation (Bolnick et al. 2003; Araújo et al. 2011; Harrison et al. 2017), spatial components of ecological niches can also vary among individuals. Whether and how these ecological consequences of inter-individual behavioral variation ultimately affect species coexistence (Chesson 2000) and maintain variation in behavioral and ecological traits and their potential covariation in natural populations will be fascinating areas of future research.

## Electronic supplementary material

Below is the link to the electronic supplementary material.

Supplementary material 1 (PDF 1620 kb)
