# Supplementary material for: Individuals in space: personality-dependent space use, movement and microhabitat use facilitate individual spatial niche specialization
Source: Oecologia. 2019 Mar 2;189(3):647–60. doi: 10.1007/s00442-019-04365-5 (PMC6418052; doi:10.1007/s00442-019-04365-5)
Supplement: Supplementary file 1 — Supplementary material 1 (PDF 1620 kb) [file 442_2019_4365_MOESM1_ESM.pdf]

Appendix: Electronic Supplement Material

Individuals in space: personality-dependent space use, movement and microhabitat use facilitate individual spatial niche specialization

Oecologia, doi: 10.1007/s00442-019-04365-5

Annika Schirmer*, Antje Herde, Jana A. Eccard, Melanie Dammhahn

Annika Schirmer, Jana A. Eccard & Melanie Dammhahn
Animal Ecology, Institute for Biochemistry and Biology
University of Potsdam, Potsdam, Germany

Antje Herde
Plant Ecology and Nature Conservation, Institute for Biochemistry and Biology
University of Potsdam, Potsdam, Germany
Department of Animal Behaviour, University of Bielefeld, Bielefeld Germany

Corresponding author:

Annika Schirmer

Animal Ecology, Institute for Biochemistry and Biology
University of Potsdam
Maulbeerallee 1, 14469 Potsdam, Germany
++49 (0)331 977 1988
annika.schirmer@uni-potsdam.de

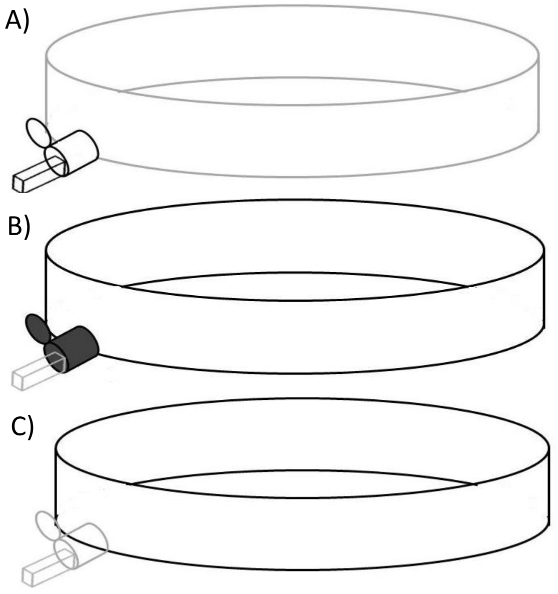

Figure A1: Setup of individual difference test. Highlighted are the respective test parts of the setup. A) Emergence test, B) Dark-Light test, C) Open field test

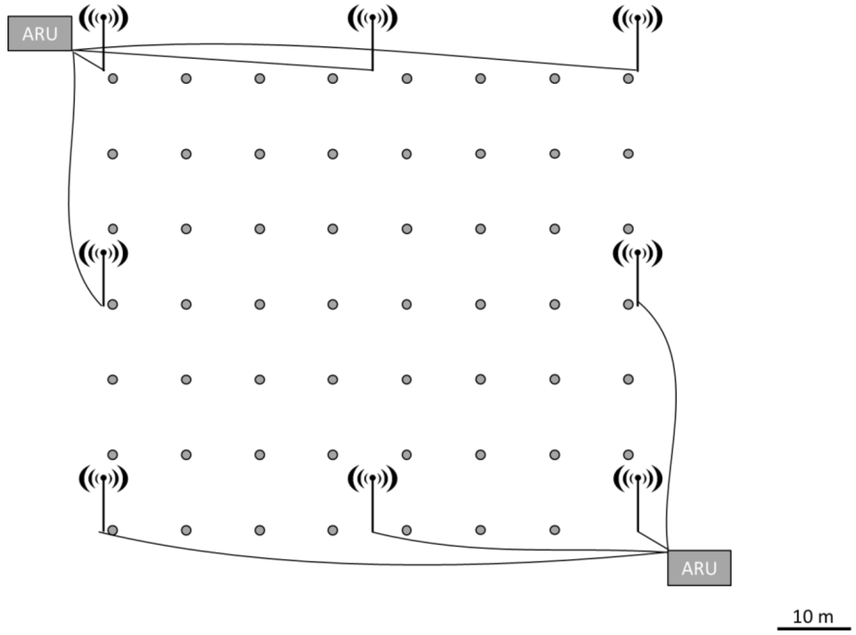

Figure A2: Schematic setup of the automated VHF telemetry. Represented are the omnidirectional antennas, the automated receiving unit (ARU, grey box) and the connecting cables (grey lines); Dots are the location of live traps of the trapping grid.

Table A1: Descriptive statistics for the studied space use and microhabitat response variables. Home ranges refer to Kernel 95 % and core areas to Kernel 50 %.

**Response**	**Mean**	**Standard deviation**	**Min**	**Max**
Home range [m^2]	2029.18	1858.88	333.46	6483.90
Core area [m^2]	549.23	467.35	89.88	1631.68
Total distance moved [m]	2892.14	1352.02	861.27	5334.69
Intraspecific home range overlap [%]	0.40	0.14	0.12	0.65
Intraspecific core area overlap [%]	0.16	0.10	0.00	0.48
Max. vegetation heigth [cm]	94.41	8.61	79.61	110.61
Ground cover [%]	50.09	6.68	42.17	70.80

Table A2: Repeatability of variables measured in the individual difference test. R represents the repeatability estimate, SE the standard error of R, CI low and CI up the lower and upper limit of the 95% -confidence interval, and the p-value for a log likelihood ratio test, testing a significant difference from zero with an assumed significance level of alpha $\leq$ 0.05. Models based on count data (numbers of crossings, sections, jumps, and activity) were calculated with a Markov-Chain-Monte-Carlo mixed model and a Poisson distribution. For these models no p-value can be obtained, since the Bayesian approach conflicts with the null hypothesis testing.

**Variable**	**R**	**SE**	**CI low**	**CI up**	**p-value**
Latency to leave the trap	-1.119	0.042	-0.211	-0.050	1.000
Latency to investigate	**0.446**	0.137	0.167	0.688	0.001
Latency to emerge	**0.372**	0.130	0.106	0.607	0.003
Latency to cross the center	**0.325**	0.144	0.001	0.581	0.007
Number of center crossings	**0.259**	0.129	0.000	0.423	NA
Number of sections entered	**0.241**	0.089	0.035	0.375	NA
Number of jumps	0.002	0.057	0.000	0.18	NA
Activity	**0.329**	0.137	0.115	0.54	NA

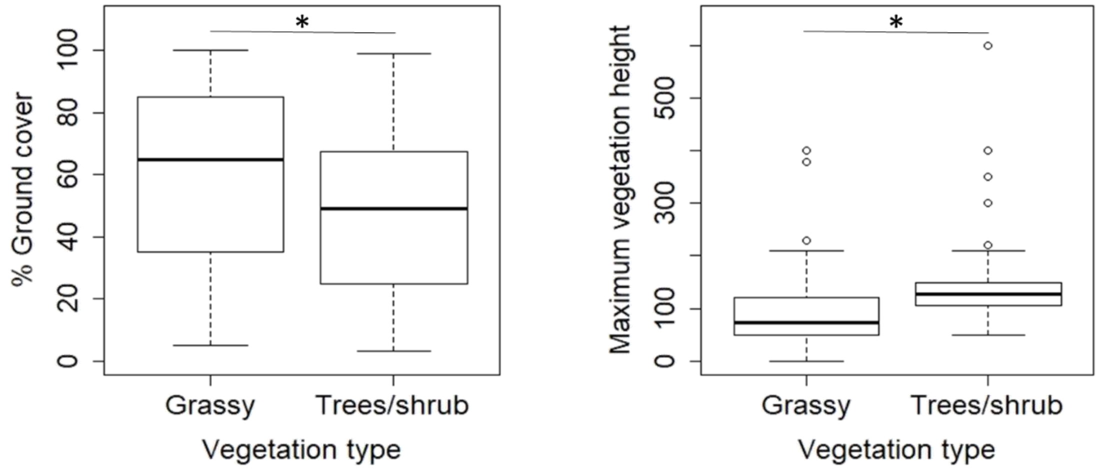

Response	Factor	Estimate	SE	t-value	χ^2	Df	p-value
Ground cover	Type	-14.25	4.23	-3.37	11.34	1	**< 0.001**
Max. Vegetation heigth	Type	69.80	15.21	4.93	21.07	1	**< 0.001**

Figure A3: Comparison of ground cover and maximum vegetation height against the dominant vegetation types. The statistical comparison shows the results of GLMMs with the factor type consisting of two levels. The grassy vegetation is thereby in the intercept and the represented values refer to the tree/shrubs. Significant differences are marked with asterisks. The graphs are based on raw data of both vegetation variables.

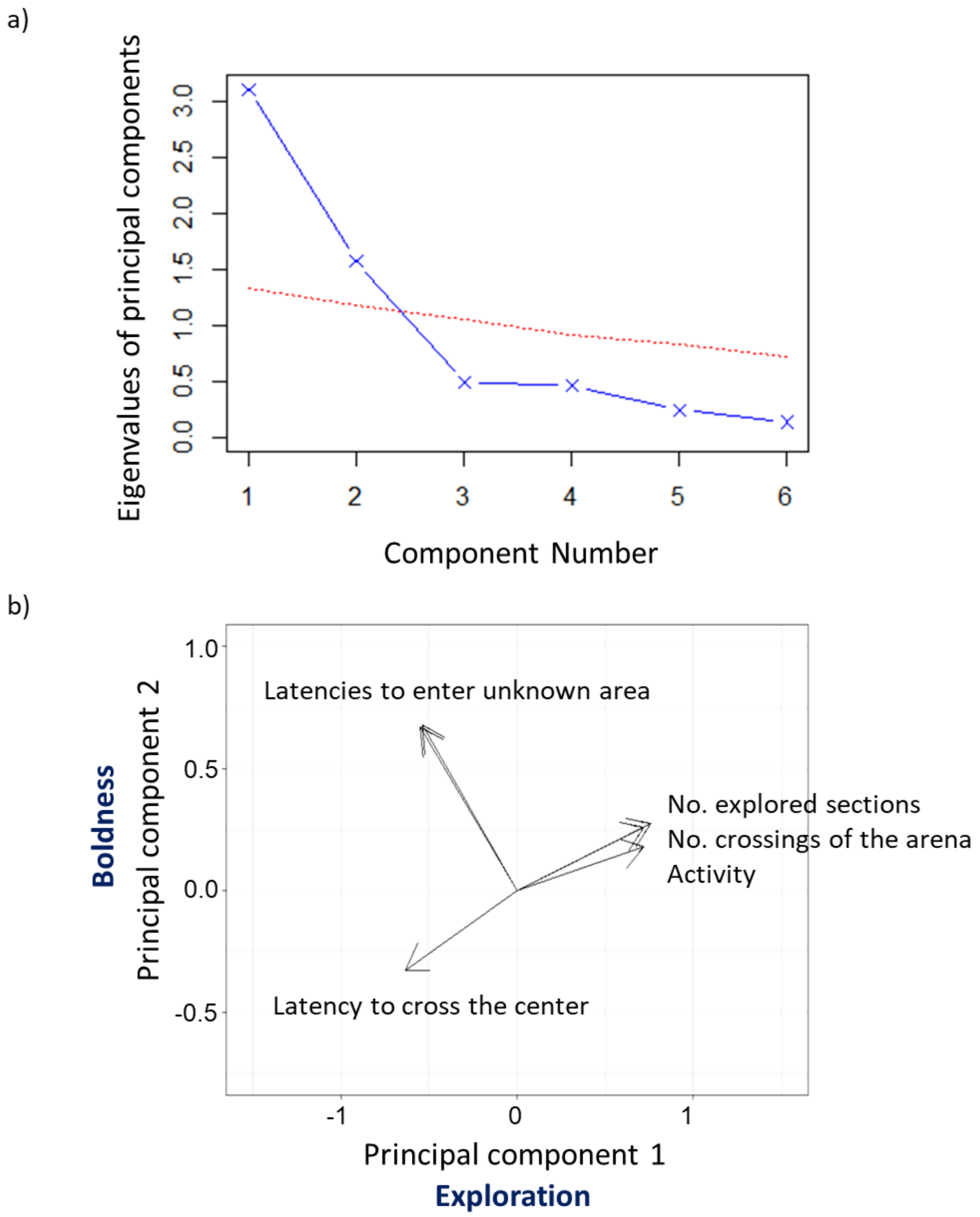

Figure A4: Components of the Principal Component Analysis (PCA). a) Screeplot of the PCA as a basis of deciding how many components to use. Represented are the actual data (blue line) and the simulated data (red dotted line). b) Division of variables on the two principal components and the obtained interpretation of scores (blue).

Table A3: Overview of PCA loadings and explained amount of variance. Latencies to investigate and emerge were inverted beforehand.

		Components	
		**PC 1**	**PC 2**
**Loadings**	Latency to investigate	-0.01	**0.96**
	Latency to emerge	0.01	**0.96**
	Latency to cross the center	**-0.81**	-0.07
	Number of center crossings	**0.82**	-0.02
	Number of sections entered	**0.89**	-0.06
	Activity	**0.83**	0.03
**Variance**	Eigenvalues	3.10	1.57
	Proportion variance	0.52	0.26
	Cumulative variance	0.52	0.78

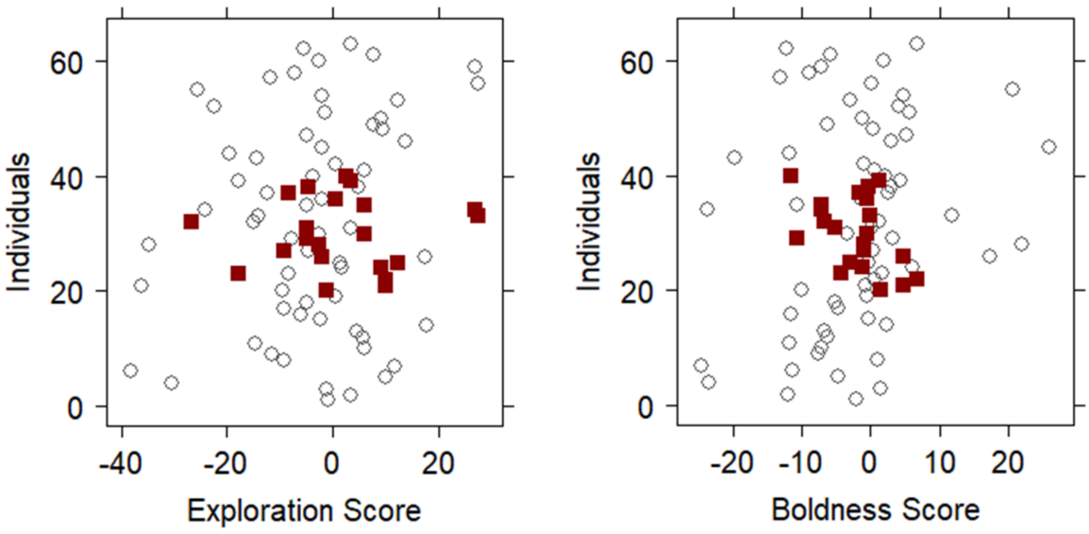

Figure A5: Distribution of exploration scores (A) and boldness scores (B) of all tested individuals (grey circles) and those tracked via VHF telemetry (red squares).

Table A4: Number of individuals tested for individual differences on the respective sites. Represented are the total numbers per site as well as separated by sex.

Site	Individuals total	Males	Females
G2	14	8	6
G3	30	18	12
G4	3	1	2
G5	8	5	3
G6	7	4	3

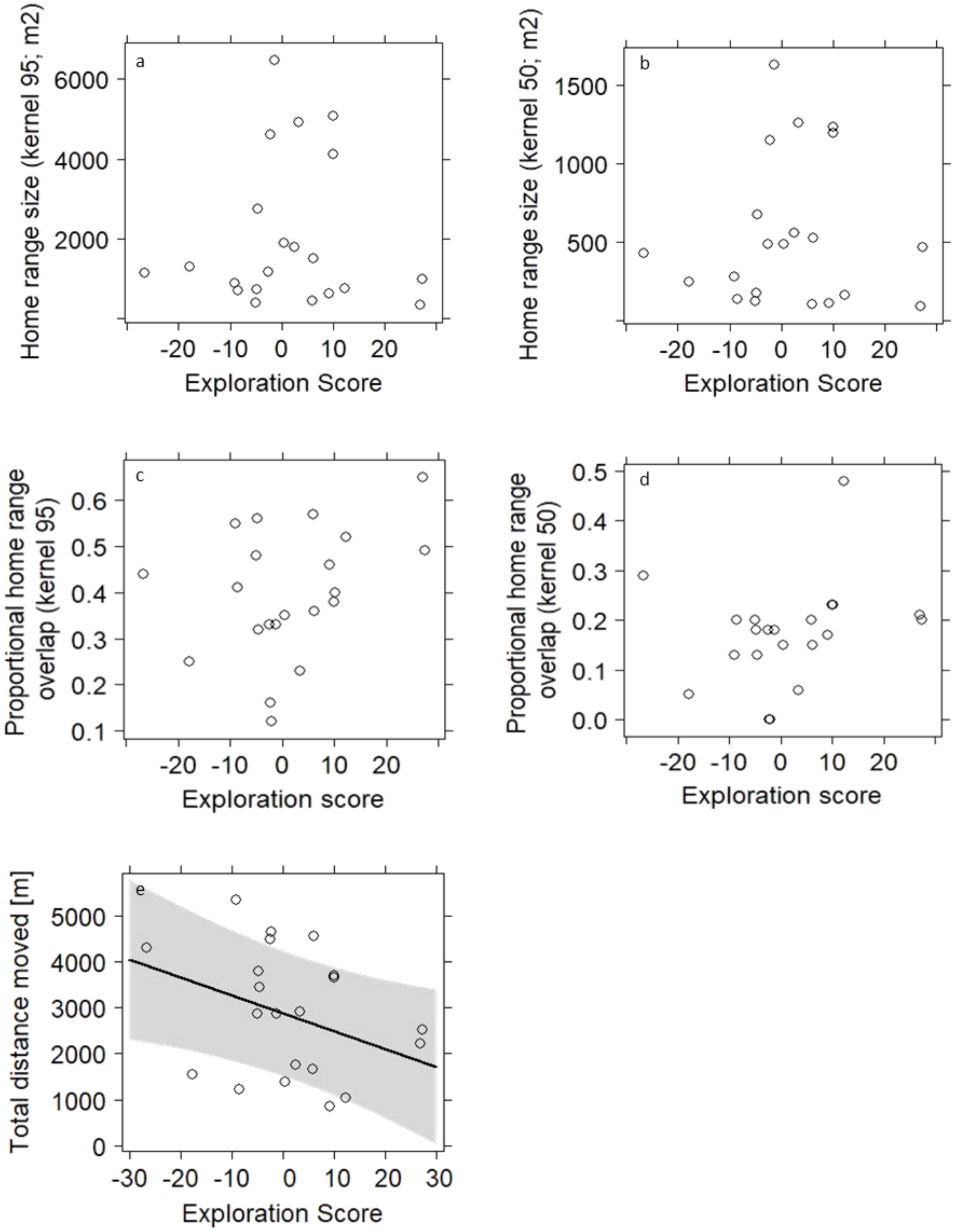

Figure A6: Effects of exploration on the home range and core area size (a, b), the spatial interactions (c, d) and the movement distance (e) of 21 radio-tracked *M. glareolus*. Represented are the effects obtained from LMMs or GLMMs (line and shaded 95%-confidence intervals) and the raw data of individuals (dots). Model effects were back transformed to the original data scale for visual representation if needed.

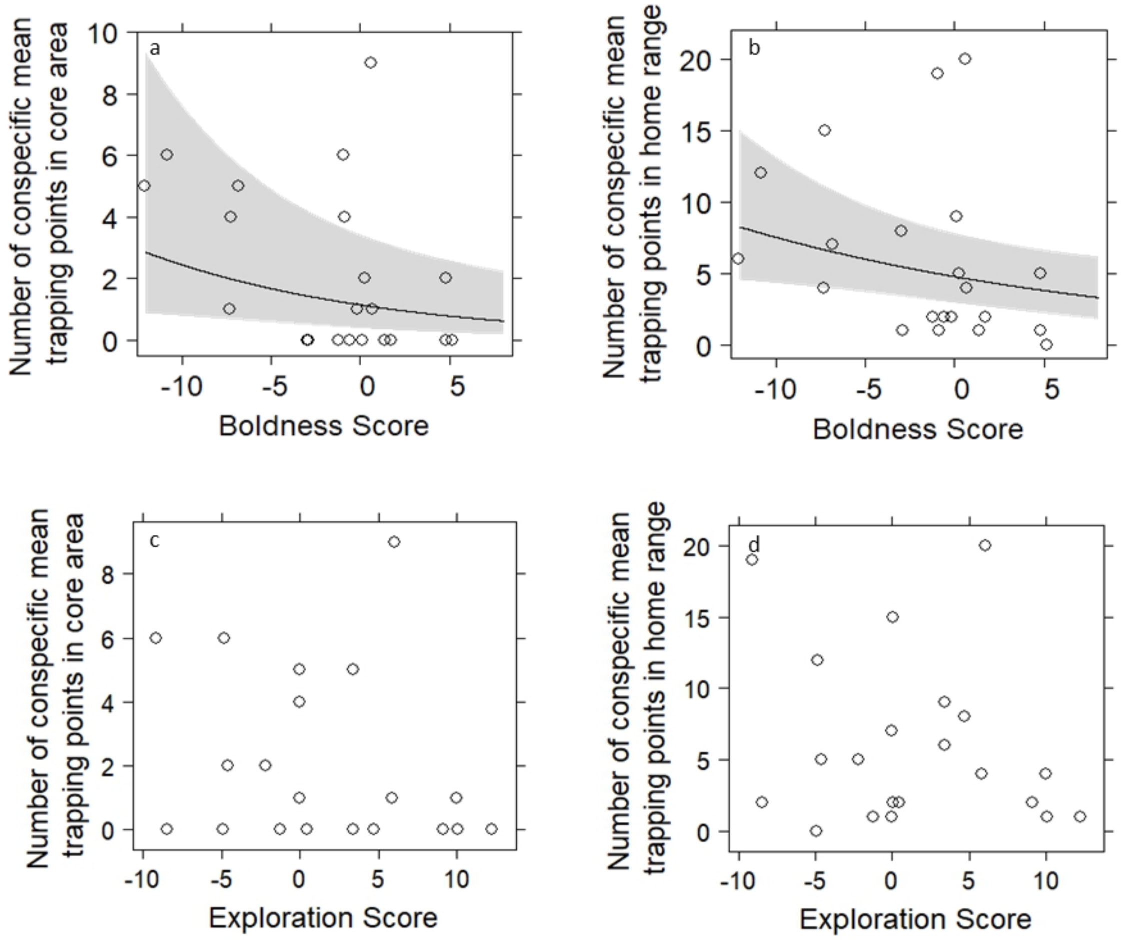

Figure A7: Effects of boldness (a, b) and exploration (c, d) on the number of mean trapping points from conspecific, residential individuals within the home ranges of 21 radio-tracked *M. glareolus*. Represented are the effects obtained from GMMs (line and shaded 95%-confidence intervals) and the raw data of individuals (dots). Model effects were back transformed to the original data scale for visual representation.

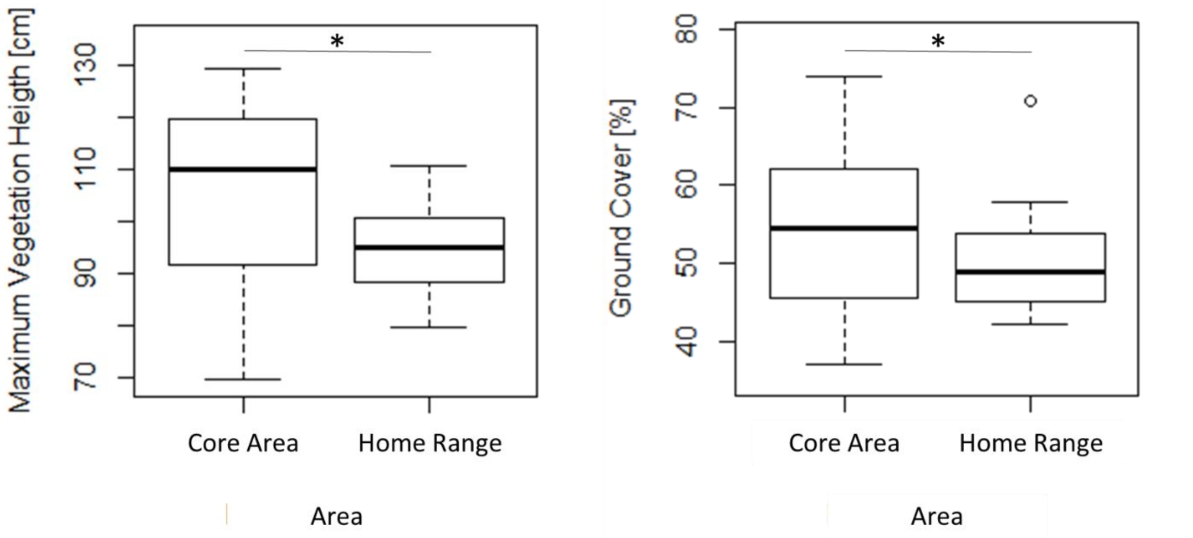

Response	Factor	Estimate	SE	t-value	χ^2	Df	p-value
Max. Vegetation heigth	Area	-10.63	39.90	3.07	-3.46	1	**< 0.001**
Ground cover	Area	-0.09	0.03	-3.13	9.79	1	**0.002**

Figure A8: Comparison of core area and home range regarding max. vegetation height and ground cover. The statistical comparison shows the results of GLMMs with the factor area consisting of two levels. The core area is thereby in the intercept and the represented values refer to the home range. Significant differences are marked with asterisks.

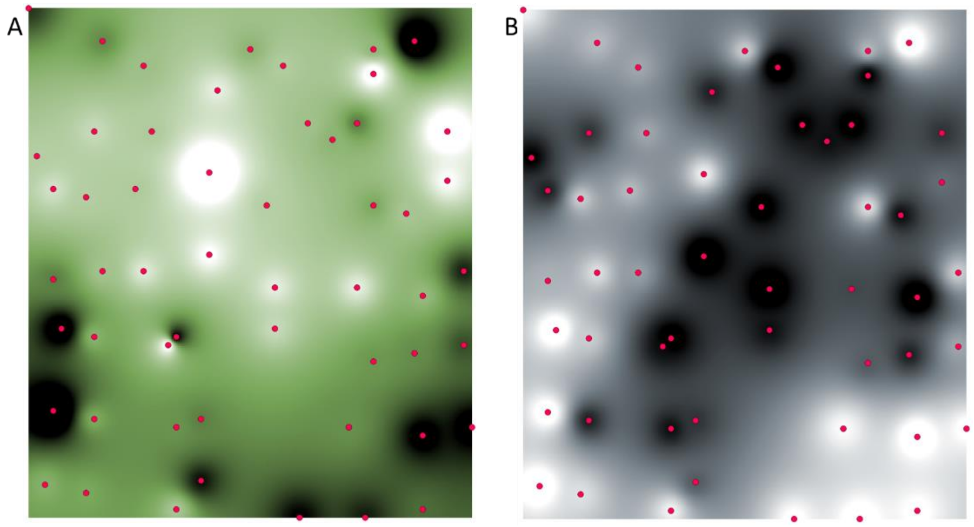

Figure A9: Exemplary interpolation of maximum vegetation height (A) and the percentage of ground cover (B) on one of the trapping grids, based on measured points on each trap location (dots). The colour gradient resembles an increase in vegetation height or percentage of ground cover from light to dark.

Table A5: Results of linear mixed models with density as an additional fixed factor to assess if differences in population densities on the study sites had an effect on the home range and core area sizes. Separate models were calculated for either of the two personality traits. Interactions between boldness and density, and exploration and density respectively, were tested but excluded via backwards model selection ($p > 0.05$).

	Variable	Estimate	SE	t-value	χ	DF	p-value
Home range size	Boldness	210.97	75.55	2.79	7.80	1	0.005
	Density	-16.66	11.66	-1.43	2.04	1	0.153
	Exploration	-14.48	25.00	-0.58	0.34	1	0.562
Core area size	Bold	58.53	19.83	2.95	8.71	1	0.003
	Density	-2.36	3.06	-0.77	0.60	1	0.440
	Explo	-2.61	6.56	-0.40	0.16	1	0.691
